# Use of Extracorporeal Membrane Oxygenation After Congenital Heart Disease Repair: A Systematic Review and Meta-Analysis

**DOI:** 10.3389/fcvm.2020.583289

**Published:** 2020-11-11

**Authors:** Yuhao Wu, Tianxin Zhao, Yonggang Li, Shengde Wu, Chun Wu, Guanghui Wei

**Affiliations:** ^1^Department of Cardiothoracic Surgery, Children's Hospital of Chongqing Medical University, Chongqing, China; ^2^Ministry of Education Key Laboratory of Child Development and Disorders, Chongqing Key Laboratory of Children Urogenital Development and Tissue Engineering, Chongqing Key Laboratory of Pediatrics, China International Science and Technology Cooperation Base of Child Development and Critical Disorders, National Clinical Research Center for Child Health and Disorders, Chongqing, China

**Keywords:** extracorporeal membrane oxygenation (ECMO), congenital heart diseases (CHD), children, outcomes, heart failure

## Abstract

**Introduction:** Extracorporeal membrane oxygenation (ECMO) has been widely used to treat cardiopulmonary failure in patients with congenital heart defects (CHD) postoperatively. A meta-analysis is performed for outcomes of postoperative CHD patients on ECMO.

**Methods:** Electronic databases, including PubMed, EMbase, and Cochrane Library CENTRAL were searched systematically from January 1990 to June 2020 for literature which reported the outcomes of postoperative CHD cases on ECMO. The scope of this search was restricted to articles published in English.

**Results:** Forty-three studies were included in this study, involving 3,585 subjects. Postoperative ventricular failure with low cardiac output was the most common indication of ECMO initiation. The pooled estimated incidence of in-hospital mortality was 56.8% (95% CI, 52.5–61.0%). Bleeding was the most common complication with ECMO with an incidence of 47.1% (95% CI, 38.5–55.8%). Multivariate meta-regression analysis revealed that single ventricular physiology (coefficient 0.213, 95% CI 0.099–0.327, *P* = 0.001) and renal failure (coefficient 0.315, 95% CI 0.091–0.540, *P* = 0.008) were two independent risk factors for in-hospital mortality.

**Conclusions:** There is an overall high in-hospital mortality of 56.8% in postoperative CHD patients on ECMO. Bleeding is the most common complication during ECMO running with an incidence of 47.1%. Single ventricular physiology and renal failure, as two independent risk factors, may potentially increase in-hospital mortality. Further studies exploring the differences in outcomes between ECMO and other extracorporeal life support strategies are warranted.

## Introduction

Although early postoperative survival after anatomic correction of Congenital Heart Diseases (CHD) has improved dramatically, there still remains a minority of children who have undergone anatomically satisfactory correction but ended up with refractory heart failure. Extracorporeal membrane oxygenation (ECMO) has been widely used to treat cardiopulmonary failure in patients with CHD after surgical repair over past decades (1, 2). ECMO remains the last resort when alternative medical treatment methods prove ineffective. In recent years, E-CPR (3), a procedure of rapid ECMO deployment, has been introduced as an immediate life support for patients having cardiac arrest but unresponsive to conventional cardiopulmonary resuscitation (CPR). However, the in-hospital mortality in connection with the use of ECMO still remains high despite significant improvements in ECMO technique and management (4). In addition, using ECMO also raises concerns over severe complications such as intracranial hemorrhage, sepsis, and renal failure (5, 6).

In current literature, mortality and morbidity of postoperative CHD patients on ECMO vary widely (5) and no consensus has been reached so far. Therefore, we perform this systematic review and meta-analysis to summarize the morbidity and mortality with its associated risk factors among patients with CHD on ECMO.

## Methods

### Literature Search

Our methods were in accordance to the Preferred Reporting Items for Systematic Reviews and Meta-Analyses (PRISMA) guidelines (7). A systematic search of the PubMed, EMbase, and Cochrane Library CENTRAL for the relevant published studies which reported the outcomes of postoperative CHD patients on ECMO was conducted in June 2020. ECMO was not commonly used for patients with CHD until the late 1980s; therefore, to avoid potential selection bias, the year of publication was restricted from January 1990 to June 2020. The search criteria were (Congenital heart diseases OR Congenital heart defects OR Congenital heart anomalies OR Congenital cardiac defects OR Congenital cardiac diseases) AND (ECMO OR ECLS OR Extracorporeal Membrane Oxygenation OR Extracorporeal Lung support OR Extracorporeal life support). References from all the included studies and other relevant literature were also reviewed to identify additional eligible studies. This search was restricted to articles published in English. We contacted the authors to obtain extra information via e-mail as necessary.

### Study Selection

A study was included in this systematic review when the following criteria were met: (1) case series, observational studies (case-controlled studies or prospective cohort studies), or randomized controlled trials (RCTs) which reported the outcomes of ECMO in postoperative CHD cases; (2) studies which reported ECMO outcomes of interest; (3) studies which included patients under 18 years of age; and (4) studies which were published on peer-review journals.

A study was excluded in this systematic review when the following criteria were met: (1) multiple studies were based on the same data; (2) sample size of the study was no more than five cases; (3) studies which involved patients diagnosed with none-CHD conditions (i.e., cardiomyopathy); (4) studies which involved patients with CHD but had ECMO institution prior to surgical repair. Reviews, letters, conference abstracts, case reports, and animal experiments were also excluded. Only the study with the most complete set of data was included when several studies were based on the same database and time period. Two reviewers (WYH and ZTX) searched and screened all the studies independently, and any disagreements on the eligibility of studies were resolved by consensus with the assistance of a third reviewer (LYG).

### Data Extraction and Definition of Variables

Data were extracted by both reviewers (WYH and WSD) independently, and any disagreement was resolved by consensus with the help of a third reviewer (WC). A standardized extraction form in an Excel spreadsheet was used. The following information was extracted: (1) baseline characteristics of the included studies: first author, publication year, study area, types of study design, types of CHD, sample size, age, chromosomal abnormalities, palliative surgery, ECMO indications, locations of ECMO cannulation (operating room, OR; intensive care unit, ICU), lowest arterial PH and peak lactate values, diagnosis of hypoplastic left heart syndrome (HLHS), cardiopulmonary bypass (CPB), and aortic clamp-cross (ACC) time, single ventricular physiology (SVP), and cannulation sites (chest vs. neck/femoral); (2) primary outcome: in-hospital mortality; and (3) secondary outcomes: late mortality in hospital survivors, duration of ECMO running, incidences of successful weaning off, residual lesions requiring reoperations, bleeding, neurological events, arrhythmia, mechanical ECMO complications, renal failure, infections, and multiple-organ dysfunction syndrome (MODS).

In-hospital mortality was defined as death before discharge. Late mortality was defined as death in hospital survivors during the follow-up. Bleeding referred to extracranial hemorrhage, hemorrhage requiring reoperation, disseminated intravascular coagulation (DIC), and any other coagulopathy. Neurological events included cognitive or motor dysfunction, seizures, and stroke which was further divided into intracranial hemorrhage and infarction. Mechanical ECMO complications referred to blood clot formation or other mechanical failure requiring circuit change. Infections were categorized into chest infection and culture-positive sepsis. Renal failure was defined as renal dysfunction requiring hemodialysis or continuous renal replacement therapy (CRRT). MODS referred to simultaneous failure of two or more organs in each individual.

### Quality Assessment and Risk of Bias

Quality assessment and risk of bias were performed by two reviewers (WYH and LYG) independently, and any divergences on the assessed quality were resolved by consensus with the assistance of a third reviewer (WC). The risk of bias of single-arm case series was evaluated with the Methodological Index for Non-Randomized Studies (MINORS) guidelines (8). The risk of bias of comparative non-RCTs was evaluated with the ROBINS-I tool (9).

### Statistical Analysis

Statistical analyses were conducted using *Stata 12.0 (Stata Corp, Texas, United States)* and Openmeta[analyst] (www.cebm.brown.edu/openmeta). The χ^2^-Q statistics and the *I*^2^ statistics were used to assess the heterogeneity, with *I*^2^ > 50% indicating significant heterogeneity. If only the median value and range were available in our included studies, the formulas provided by Hozo et al. (10) would have been used to estimate the mean value. A *p* < 0.05 was considered statistically significant for all analyses in our study.

For single-arm case series, we performed a meta-analysis of proportions to pool postoperative morbidity. We utilized a random-effect model for meta-analysis due to the likelihood of inter-observational heterogeneity. The Freeman–Tukey Double Arcsine Transformation was used to adjust proportions. Adjusted estimates were pooled by the generic inverse variance method. Subgroup and meta-regression analyses were used to explore risk factors associated with the primary outcome. Subgroup analysis was conducted in terms of the year of publication, study area, age, weight, SVP, HLHS, locations of cannulation, ECPR, bleeding, renal failure, MODS, and ECMO duration. Meta-regression analysis was performed with random models using aforementioned continuous variables.

For case-controlled studies, we performed a meta-analysis to evaluate the outcomes of two compared interventions. Odds ratio (OR) was employed in this setting. If the *I*^2^ statistic was over 50%, a random-effect model was adopted; otherwise, a fixed-effect model of analysis was conducted.

## Results

### Characteristics of Included Studies

**Figure 1:**
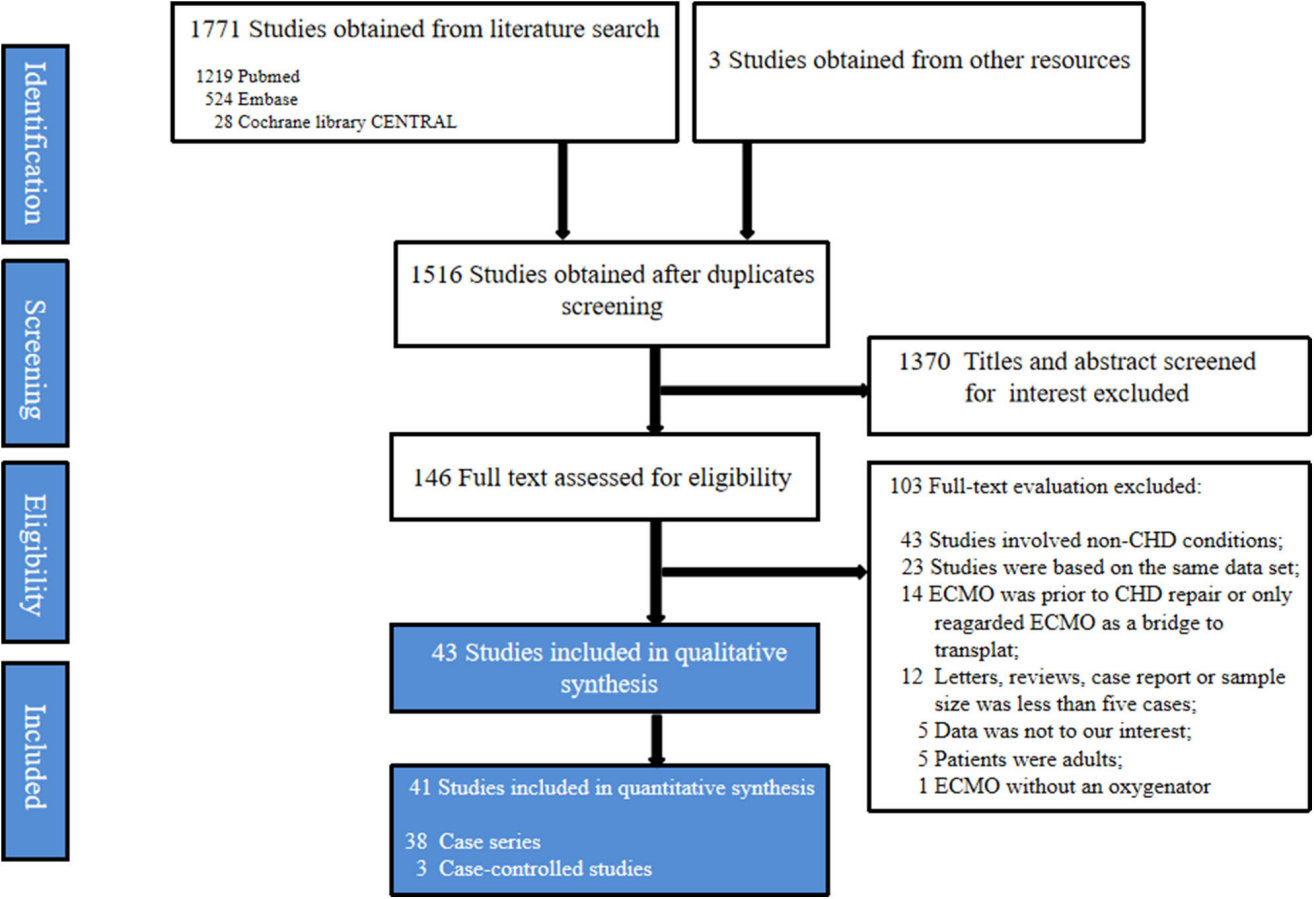
Flow diagram according to the preferred reporting items for systematic review and meta-analysis (PRISMA) protocol recommendations. ECMO, Extracorporeal membrane oxygenation; CHD, Congenital heart diseases.

A total of 1,771 studies were obtained initially from the electronic databases, and 3 studies were further identified manually on the reference lists of the retained studies. After screening for duplicates and relevance in titles and abstracts, only 146 studies were available for the full-text evaluation. One hundred and three studies were excluded after full-text evaluation. Eventually, this systematic review was based on 43 studies, encompassing 38 case series (11–48) and 5 case-controlled studies (49–53). A flowchart depicting the search strategy is shown in the Figure 1.

A total of 3,585 patients were involved in this study (Supplementary Table 1). Although three studies (38, 40, 49) were performed by Alsoufi et al. these studies were based on data from different institutions. Therefore, they were considered as independent studies and included in this research. Among our included studies, two studies (35, 41) were originated from the Extracorporeal Life Support Organization (ELSO) registry and the Pediatric Health Information System (PHIS) databases, respectively. Nine studies (23, 25, 35, 36, 40, 42, 44, 49, 53) were completely based on the neonate population, and most of their patients underwent palliative surgery before ECMO initiation.

ECMO was most commonly performed in children undergoing the Norwood palliative operation for HLHS and complex biventricular repair (Supplementary Table 2). SVP was observed in over one-third of our included patients. As compared with peripheral cannulation, which might be a preferred approach to reduce the risks of bleeding and infection, central cannulation involving atrium and aorta was adopted more frequently. The most common indication for ECMO initiation was postoperative ventricular failure with low cardiac output (LCO). ECMO initiation due to pulmonary artery hypertension, failure to wean from CPB, cardiac arrest, and pulmonary failure were also present in different proportions (Supplementary Table 1). However, ECMO was rarely adopted in patients with residual lesions (46) and shunt obstructions (42, 45, 50). ECPR, a rapid ECMO deployment procedure replacing conventional CPR, was only performed in 618 patients. Four studies reported the successful use of veno-venous ECMO when patients had an adequate cardiac function and needed lung support (29, 34, 35, 47). Ventricular venting was only reported in 6 studies (11, 13, 15, 18, 19, 31). Bridging to heart transplant was reported in seven studies (12, 13, 27, 31, 34, 35, 53), while a subsequent ventricular assist device (VAD) implantation was only reported in one study (50). A total of 11 studies (12, 14, 16, 23, 24, 26, 36, 38, 44, 45, 48) reported the causes to in-hospital mortality, which was mainly caused by MODS, infections, and ventricular failure.

In addition, three case-controlled studies (49, 50, 52) compared VAD with ECMO. One study (51) compared conventional priming with mini-priming strategies. One study (53) compared clinical outcomes in patients with or without ECMO usage after the Norwood procedure.

### Risk of Bias

Due to the lack of blind evaluation of endpoints, follow-up data, and prospective calculation of the sample size, MINORS scores of 22 case series were lower than 10 points. The RONBINS-I tool revealed a moderate risk of bias in all five case-controlled studies.

### Primary Outcome

#### In-Hospital Mortality

**Figure 2:**
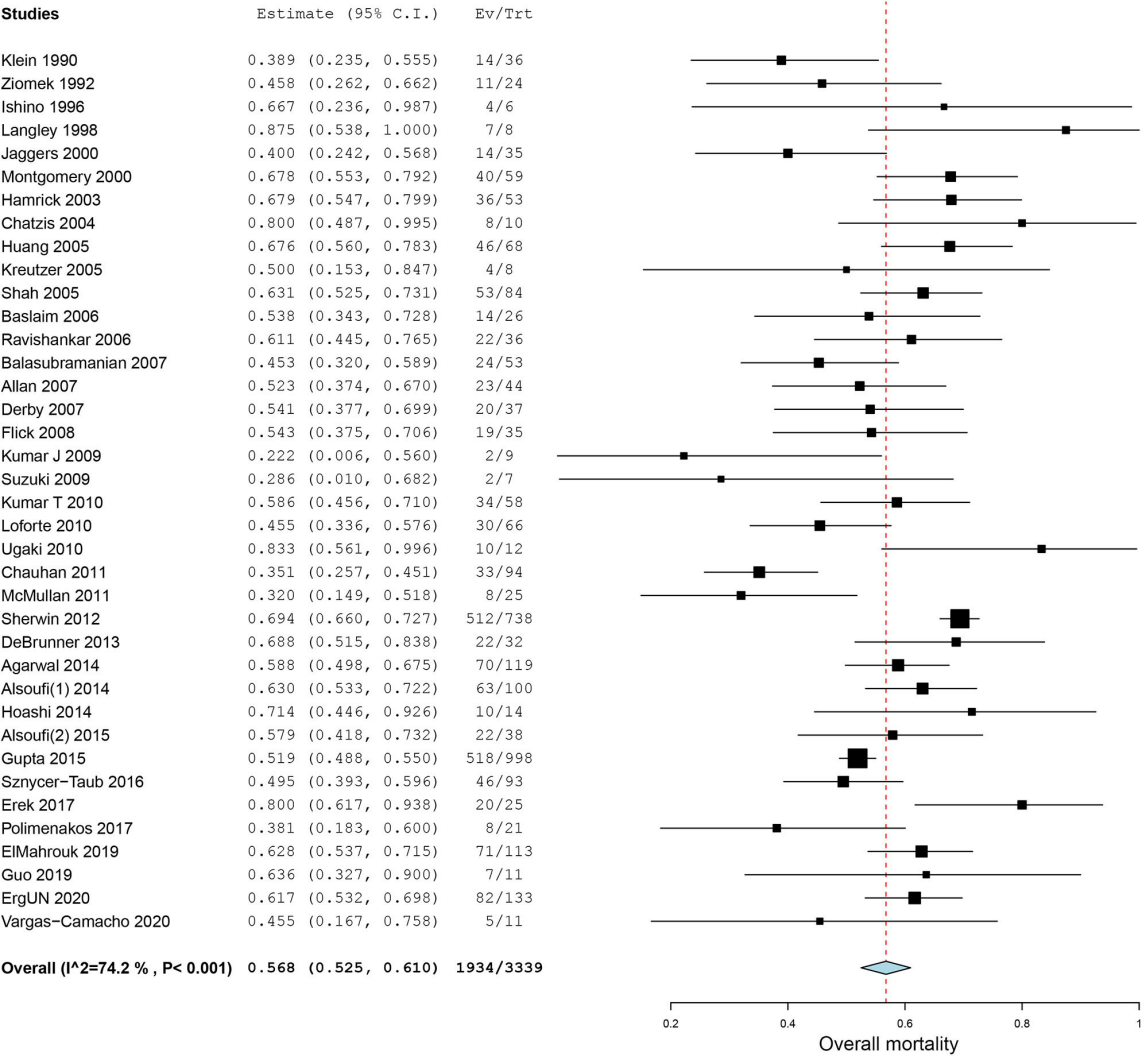
Forest plot of in-hospital mortality for patients receiving extracorporeal membrane oxygenation after congenital heart diseases surgery.

All of our included case series reported in-hospital mortality (Figure 2). Using a random-effect model, the pooled estimate of in-hospital mortality was 56.8% (95% CI, 52.5–61.0%, *I*^2^ = 74.2%, Figure 2).

### Secondary Outcomes

#### Incidence of Successful Weaning Off

A total of 30 case series reported successful weaning off in 1,199 patients. Using a random-effect model, the pooled estimate of the incidence of successful weaning off was 60.3% (95% CI, 57.0–63.7%, *I*^2^ = 35.4%, Supplementary Figure 1).

#### Late Mortality in Hospital Survivors

A total of 16 case series reported late mortality in hospital survivors during the follow-up. The pooled estimate of late mortality was 20.5% (95% CI, 13.5–28.2%, *I*^2^ = 11.0%, Supplementary Figure 2). Furthermore, seven studies (14, 15, 18, 23, 29, 43, 44) reported that all late deaths occurred within 1 year after discharge. Jaggers et al. (15) and DeBrunner et al. (36) reported a total of 4 late deaths between 1 and 2 years after discharge. Balasubramanian et al. (24) reported late mortality of 31% after a mean follow-up of 75 months. Overall survival following Norwood palliation in (40) cohort was 67% at 5 years.

#### Residual Lesions Requiring Reoperations

A total of 16 case series reported residual lesions requiring reoperation in 330 patients. The pooled estimate of residual lesions requiring reoperations was 14.9% (95% CI, 9.8–20.7%, *I*^2^ = 80.3%, Supplementary Figure 3).

#### Adverse Events

**Figure 3:**
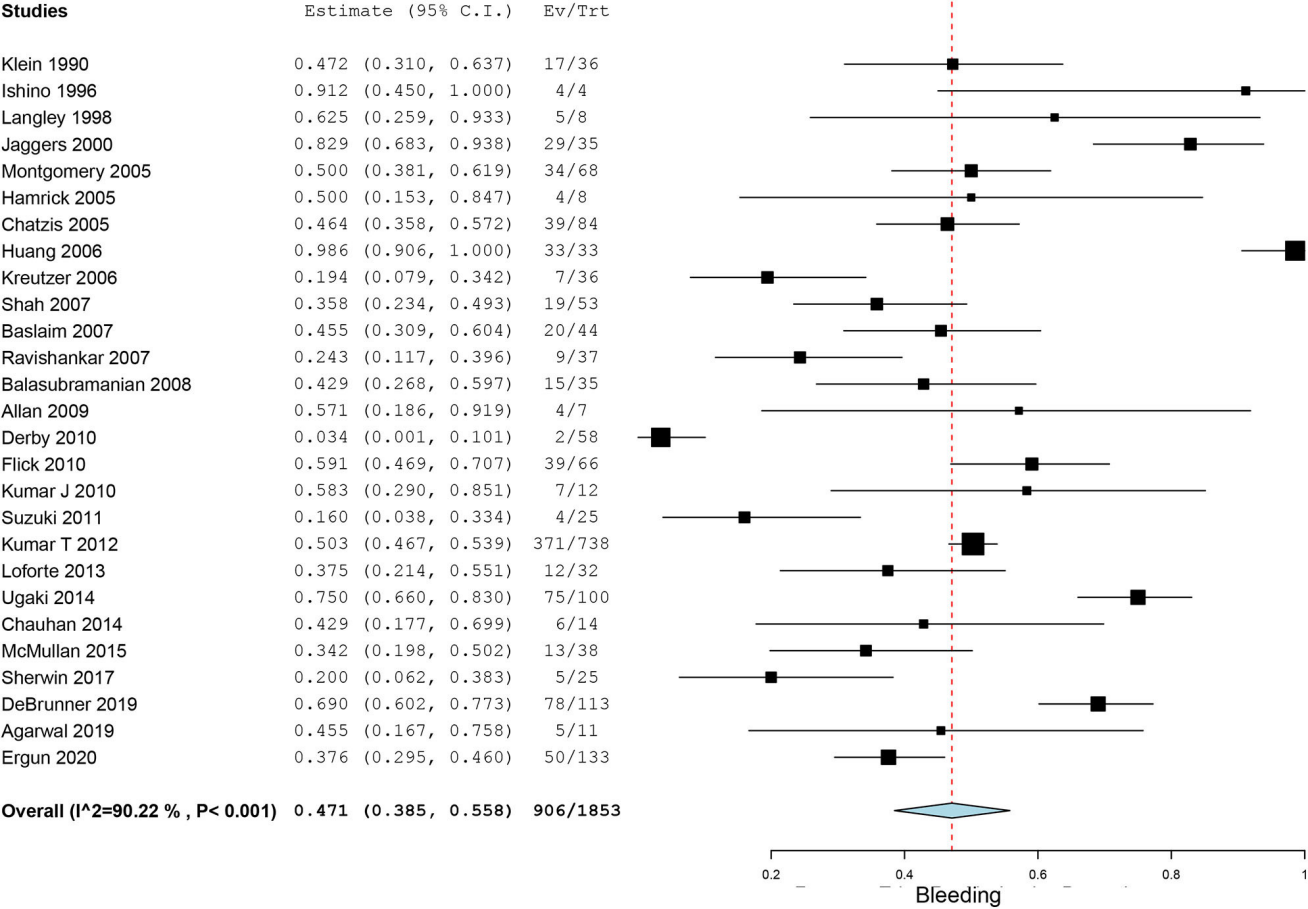
Forest plots assessing incidence of bleeding in patients on extracorporeal membrane oxygenation.

**Figure 4:**
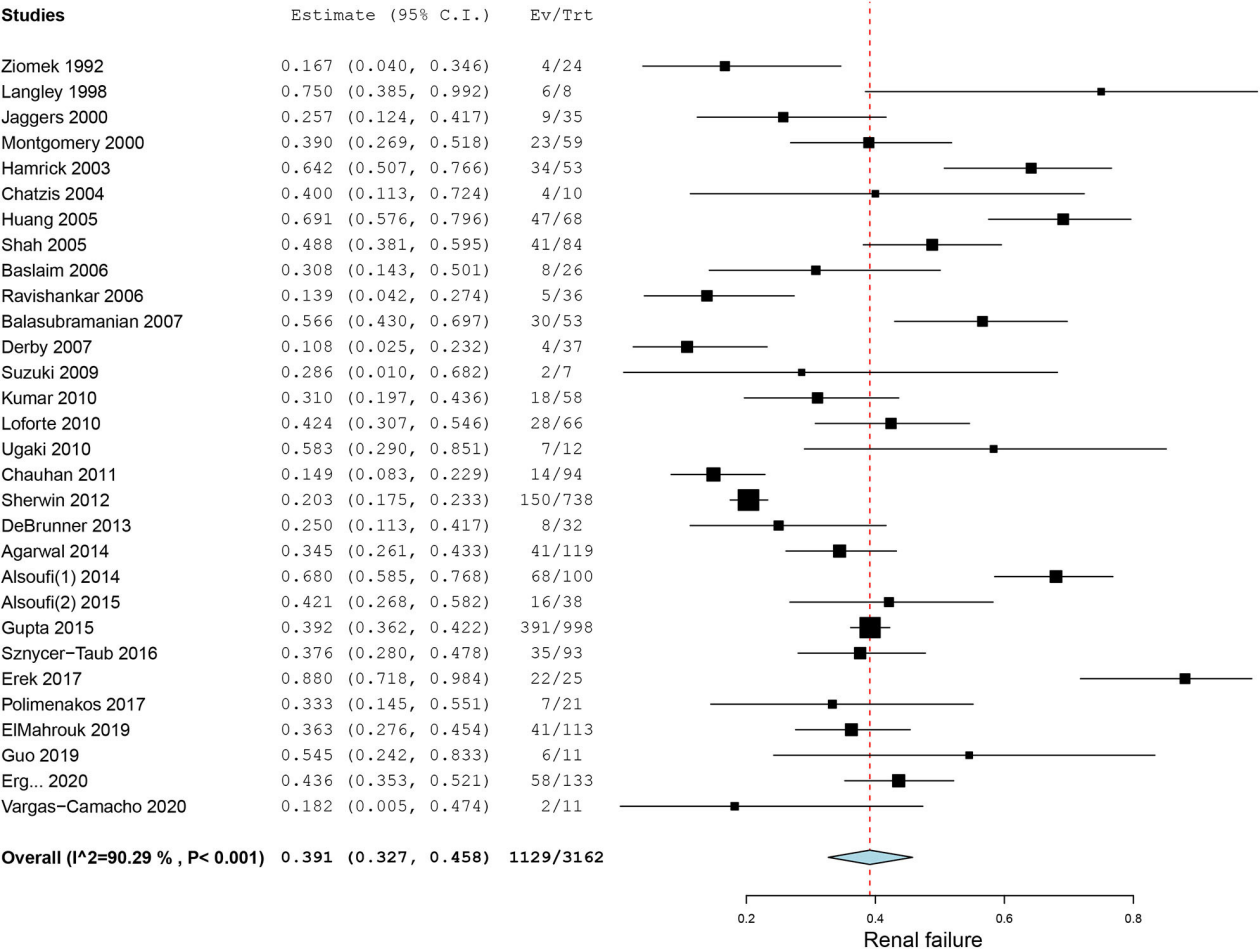
Forest plot of incidence of renal failure in patients on extracorporeal membrane oxygenation after congenital heart diseases surgery.

Bleeding, a common complication during ECMO running, was reported in 27 case series. The pooled incidence of bleeding was 47.1% (95% CI, 38.5–55.8%, *I*^2^ = 90.2%, Figure 3). Neurological events of patients on ECMO were reported in 23 case series. Particularly, we also observed seizures and cognitive and motor dysfunction in 13 patients after discharge. The pooled overall incidence of neurological events was 16.1% (95% CI, 11.0–21.9%, *I*^2^ = 90.0%, Supplementary Figure 4). Incidence of ECMO mechanical complications was recorded in 16 case series with an incidence of 18.8% (95% CI, 9.9–29.5%, *I*^2^ =94.2%, Supplementary Figure 5). Renal failure was reported in nearly one-third of our included patients. The pooled estimated incidence of renal failure was 39.1% (95% CI, 32.7–45.8%, *I*^2^ = 90.3%, Figure 4). Sepsis, a strong indicator for in-hospital mortality, was extracted from our included studies independently. The pooled estimated incidence of systemic sepsis was 16.7% (95% CI, 10.0% to 24.6%, I^2^ =93.4%, Supplementary Figure 6). The pooled estimated incidence of MODS was 19.0% (95% CI, 10.7–29.0%, *I*^2^ = 96.0%, Supplementary Figure 7). Only 9 studies reported respiratory complications, and the pooled estimated incidence of respiratory complications was 15.5% (95% CI, 7.5–25.3%, *I*^2^ = 86.2%, Supplementary Figure 8).

#### Subgroup Analysis

We further applied subgroup analysis to in-hospital mortality (Table 1). Subgroup analysis was conducted in terms of year of publication, study area, age, weight, SVP, locations of cannulation, ECPR, bleeding, renal failure, MODS, HLHS, and ECMO duration. In-hospital mortality was highest in the 1990s, as compared with the other two time periods. Younger patients entailed higher in-hospital mortality than those of older age. Patients weighed <5 kg entailed the highest in-hospital mortality. ECMO initiation in the OR and longer duration of ECMO running were also associated with higher in-hospital mortality. As the incidences of bleeding, SVP, ECPR, ECMO initiation due to failure to wean from CPB, renal failure, HLHS, and MODS increased, so did in-hospital mortality.

**Table 1:** Subgroup analysis of in-hospital mortality.

**Subgroups**	**Studies (*n*)**	**Pooled estimates of in-hospital mortality (95% CI)**	**Heterogeneity (*I^**2**^ %*)**
**Year of publication**			
1990–1999	4	58.2% (35.0–81.3%)	76.4
2000–2009	15	56.4% (50.1–62.6%)	55.0
2010–2020	19	57.6% (51.9–63.3%)	84.9
**Study area**			
Asia	11	58.7% (48.4–69.0%)	80.2
Europe	4	62.1% (42.5–81.8%)	81.2
North America	21	55.4% (50.3–60.6%)	78.9
Other	2	62.0% (53.4–70.6%)	0
**Average or median age**			
≤ 1 month	13	59.5% (53.1–66.0%)	83.8
1 month to 1 year	16	56.6% (49.1–64.1%)	78.2
≥1 year	6	45.3% (37.8–52.9%)	0
**Weight**			
≤ 5 kg	23	57.6% (52.6–62.5%)	81.5
5 to 10 kg	7	52.9% (38.9–66.8%)	74.7
≥10 kg	2	48.3% (34.3–62.2%)	9.3
**Incidence of SVP**			
≤ 50%	20	52.8% (47.5–58.1%)	65.2
>50%	11	61.6% (55.0–68.1%)	59.6
**Incidence of HLHS**^***&***^			
Reported	19	59.8% (54.3–65.2%)	66.2
None reported	19	54.4% (49.0–59.8%)	72.8
**Incidence of cannulation at OR**			
≤ 50%	12	57.1% (51.7–62.6%)	49.2
>50%	9	57.3% (44.2–70.4%)	84.0
**Incidence of bleeding**			
≤ 50%	15	57.4% (51.7–63.1%)	53.8
>50%	12	61.2% (53.9–68.4%)	71.9
**Incidence of renal failure**			
≤ 50%	22	55.6% (50.5–60.7%)	81.3
>50%	8	68.2% (59.5–76.9%)	62.7
**MODS**^&^			
Reported	19	60.0% (54.3–65.8%)	83.8
None reported	19	53.2% (47.3–59.1%)	60.3
**Mean ECMO duration**			
≤ 72 h	3	39.0% (23.3–54.7%)	63.5
72 to 144 h	22	56.7% (51.5–61.8%)	80.6
≥144 h	10	64.2% (56.7–71.7%)	45.4
**ECMO initiation due to ventricular failure**			
≤ 50%	19	58.3% (53.0–63.5%)	70.8
>50%	12	54.3% (44.7–64.0%)	77.4
**ECMO initiation due to failure to wean from CPB**			
≤ 50%	28	56.3% (51.1–60.5%)	78.1
>50%	3	58.3% (40.5–76.1%)	72.3
**Incidence of ECPR**			
≤ 50%	18	58.1% (52.5–63.8%)	80.7
>50%	10	58.9% (50.7–67.1%)	58.8

SVP, Single ventricular physiology; OR, Operating room; ECPR, Extracorporeal cardiopulmonary resuscitation; MODS, Multiple-organ dysfunction syndrome; ECMO, Extracorporeal membrane oxygenation; HLHS, Hypoplastic left heart syndrome; CPB, Cardiopulmonary bypass.

& *Since the MODS and HLHS were not commonly reported, therefore we only divided these outcomes into reported and none-reported subgroups*.

#### Meta-Regression Analysis

**Figure 5:**
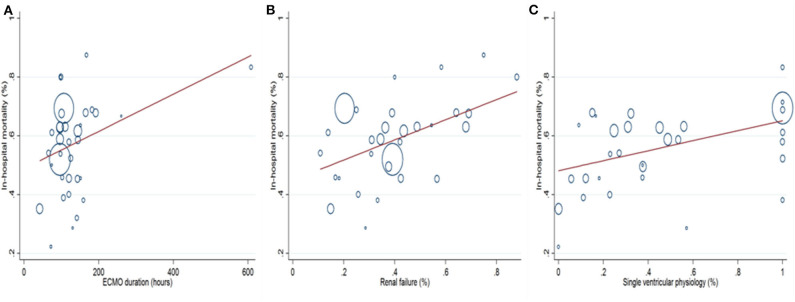
Univariate meta-regression analysis shows that prolonged ECMO duration **(A)**, increased incidences of renal failure **(B)**, and single ventricular physiology **(C)** are associated with higher in-hospital mortality.

Prespecified variables, which were relevant to in-hospital mortality, were collected. The variables included CPB, ACC, cannulation at OR, renal failure, stroke, bleeding, ECPR, age, weight, peak lactate, lowest arterial PH, SVP, HLHS, ECMO duration, sepsis, and MODS. Univariate meta-regression analysis (Supplementary Table 3) identified SVP (coefficient 0.169, 95% CI 0.053–0.286, *P* = 0.006), renal failure (Coefficient 0.343, 95% CI 0.117–0.568, *P* = 0.004), and ECMO duration (coefficient 0.001, 95% CI 0.000–0.001, *P* = 0.031) as the risk factors for in-hospital mortality (Figure 5). A further multivariate meta-regression revealed that SVP (coefficient 0.213, 95% CI 0.099–0.327, *P* = 0.001) and renal failure (coefficient 0.315, 95% CI 0.091–0.540, *P* = 0.008) were the only two independent risk factors for in-hospital mortality.

#### ECMO vs. Ventricular Assist Devices (VAD)

Three case-controlled studies compared in-hospital mortality between VAD and ECMO in patients following CHD repair. No significant difference was found between these two strategies (OR, 1.628; 95% CI, 0.674–3.932; *P* = 0.278; *I*^2^ = 78.4%).

## Discussion

To our knowledge, this is the first meta-analysis evaluating outcomes of patients with CHD on ECMO. In this study, we found that postoperative ventricular failure with LCO, failure to wean from CPB, pulmonary artery hypertension, pulmonary failure, and cardiac arrest were crucial indicators for ECMO institution. Routine institution after Norwood palliative procedures for HLHS was also an indicator for ECMO usage (54, 55). Although 60.3% of patients receiving ECMO after CHD surgery successfully weaned from ECMO; only 43.2% of patients survived to discharge. Late mortality, which was less likely related to ECMO usage, occurred in 21.7% of hospital survivors. Since only two studies reported follow-up data over 5 years, the actual mortality of hospital survivors in long-term follow-up remained undetermined.

Residual lesions, which were related to hemodynamic instability and difficulty in weaning from ECMO, were generally found through echocardiogram or catheter-based diagnostic studies in patients on ECMO, and these lesions were repaired by either surgery or catheter-based procedures in 15.3% of our involved patients. Catheterization should be performed when an echocardiogram failed to find a reason for failure to wean from ECMO (56). Bleeding, the most common complication in our study, was observed in almost half of involved patients. Renal failure, which was also a strong indicator for in-hospital mortality (22), occurred in over one-third of the involved patients. Neurological events, encompassing stroke, seizures, cognitive, or motor dysfunction, were found in one-sixth of involved patients. Sepsis and mechanical ECMO complications were observed in 17.5 and 22.6% of included patients, respectively.

To explore the risk factors for in-hospital mortality, subgroup and meta-regression analyses were performed. We found that in-hospital mortality has decreased in a chronological manner. This was probably due to great improvements in surgical technique, intensive care, and ECMO management in past decades. Surgical repair of CHD at a young age was generally connected to significant mortality, and we were not surprised that the neonates' subgroup in this research entailed the highest in-hospital mortality. Patients with lower body weight were also associated with higher mortality. In addition, ECMO initiation at the OR, longer ECMO duration, ECMO initiation due to failure to wean from CPB, increased incidences of ECPR, SVP, bleeding, renal failure, HLHS, and MODS were all associated with higher in-hospital mortality (Table 1). ECMO initiation at the OR was mainly due to failure to wean from CPB. Patients in such conditions might have sustained significant myocardial damage during operations and thus were less likely to survive despite ECMO support. ECMO durations of 72 and 144 h were used as cutoff times in this study. In the literature (16, 22, 23), ECMO running longer than these cutoff times was associated with drastic reductions in survival. We found a similar trend in this regard where ECMO duration longer than 144 h entailed the highest mortality (64.2%). However, only three risk factors were statistically associated with in-hospital mortality in the univariate meta-regression analysis (Supplementary Table 3). Remarkably, SVP (*P* = 0.006), renal failure (*P* = 0.004), and prolonged ECMO duration (*P* = 0.031) increased in-hospital mortality in a positive correlation. A further multivariate meta-regression analysis confirmed SVP (*P* = 0.001) and renal failure (*P* = 0.008) as two independent risk factors for in-hospital mortality. Additionally, no difference was found between ECMO and VAD in postoperative patients with CHD on ECMO regarding in-hospital mortality.

Although the inception of ECMO allows surgeons to perform a corrective repair in patients at extreme risk, survival is more likely when underlying conditions are reversible (11, 14). Reoperation to correct residual lesions (i.e., graft obstruction) is crucial for weaning from ECMO. Early use of echocardiogram to ascertain the adequacy of surgical repair before instituting ECMO is recommended (14, 37). The American Heart Association recommends (57) catheterization for early intervention when there are suspicions for residual lesions. Fatal complications, contributing to in-hospital mortality, can also be prevented and reversed. Bleeding can be controlled with close monitoring of Activated Clotting Times (ACT), proper use of blood products, the introduction of a heparin-coated circuit, and meticulous surgical hemostasis (12). Renal failure and MODS are generally related to critical LCO; therefore, timely ECMO rescue and sufficient flow before the onset of organ failure may prevent and reverse the development of end-organ damage (15). To shorten the “set-up” time of ECMO institution, a procedure for rapid deployment (i.e., pre-primed circuit and rapid-response team) should be considered (22, 32, 44). In patients with borderline hemodynamics, delayed chest closure facilitates chest cannulation and open cardiac massage and also avoids interruption of CPR. However, this also simultaneously increases potential risks of major bleeding, infection, and sepsis, especially in patients with prolonged use of ECMO. Therefore, aggressive prevention of sepsis is warranted in such scenarios. New strategies to alleviate the inflammatory effects of ECMO may be helpful in reducing MODS and sepsis (30, 58).

Based on the current study, the optimal timing for the cessation of ECMO running cannot be determined in patients who are lack of myocardial recovery after a prolonged ECMO running. Moreover, we are not capable to evaluate and discuss ethical problems such as discontinuing ECMO when survival is not expected or with high risks of mortality or poor outcomes. ECMO usage is associated with high mortality, which results in ethical challenges and in turn creates conflicts within an ECMO team or between a team and the family (59). Further investigations and discussions should be conducted on how to compassionately discontinue an unsuccessful ECMO institution.

We acknowledged the limitations of our study here. First, our study was mainly based on retrospective studies, and hence our meta-analysis was of limited quality. Second, we could not make a conclusion as to whether shunt should be left open on ECMO in shunt-dependent pulmonary circulation due to limited data. Although maintaining shunt patency during ECMO support can reduce the risk of pulmonary ischemia–reperfusion injury and shunt thrombosis (60), this also allows the drainage of excessive diastolic flow and reduces coronary circulation. Third, the ventricular venting strategy could not be evaluated in this study either due to inconsistent reports from our included studies. Finally, the actual incidence of neurological events is difficult to identify if patients die while still on ECMO, and it is also difficult to assess whether these abnormalities are attributed to ECMO directly. Moreover, cognitive or motor dysfunction is reported in short-term follow-up. Long-term neurological conditions and abnormalities are not given in our included studies; therefore, the impact of ECMO on psycho-dynamic growth and performances cannot be determined based on current research.

## Conclusions

In conclusion, postoperative ventricular failure with LCO is the most common indication for ECMO institution in postoperative patients with CHD. There is an overall high in-hospital mortality of 56.8%, although 60.3% of patients successfully wean from ECMO. Bleeding is the most common complication during ECMO running with an incidence of 47.1%. SVP and renal failure, as two independent risk factors, may potentially increase risks of in-hospital death. However, the amount of evidence from current research is limited due to our retrospective nature. More RCTs should be designed to explore the differences in clinical outcomes between ECMO and other extracorporeal life support strategies.

## Data Availability Statement

The raw data supporting the conclusions of this article will be made available by the authors, without undue reservation.

## Author Contributions

YW and TZ: project development, data collection, and manuscript writing. YL and SW: data collection. YW and CW: manuscript writing. GW and CW: critical revision. All authors contributed to the article and approved the submitted version.

## Conflict of Interest

The authors declare that the research was conducted in the absence of any commercial or financial relationships that could be construed as a potential conflict of interest.

## Supplementary Material

Supplementary Figure 1 Forest plot of incidence of successful weaning off in patients on extracorporeal membrane oxygenation.

Supplementary Figure 2 Forest plot of incidence of late mortality in hospital survivors.

Supplementary Figure 3 Forest plot of incidence of residual lesions requiring reoperations in patients on extracorporeal membrane oxygenation.

Supplementary Figure 4 Forest plot of incidence of neurological events in patients on extracorporeal membrane oxygenation.

Supplementary Figure 5 Forest plot of incidence of ECMO mechanical complications in patients on extracorporeal membrane oxygenation.

Supplementary Figure 6 Forest plot of incidence of sepsis in patients on extracorporeal membrane oxygenation.

Supplementary Figure 7 Forest plot of incidence of MODS in patients on extracorporeal membrane oxygenation.

Supplementary Figure 8 Forest plot of incidence of respiratory complications in patients on extracorporeal membrane oxygenation.

Supplementary Table 1 Baseline characteristics of included studies.

Supplementary Table 2 Types of involved congenital heart diseases and single ventricular physiology.

Supplementary Table 3 Meta-regression analysis for in-hospital mortality based on pre-specified patients' variables.

## Abbreviations

ACC: Aortic Cross-Clamp
ACT: Activated Clotting Times
CHD: Congenital Heart Diseases
CPB: Cardiopulmonary Bypass
CPR: Cardiopulmonary Resuscitation
CRRT: Continuous Renal Replacement Therapy
DIC: Disseminated Intravascular Coagulation
ECLS: Extracorporeal life support
ECMO: Extracorporeal Membrane Oxygenation
ECPR: ECMO-Cardiopulmonary Resuscitation
ELSO: Extracorporeal Life Support Organization
HLHS: Hypoplastic Left Heart Syndrome
ICU: Intensive Care Unit
LCO: Low Cardiac Output
MINORS: Methodological Index for Non-randomized Studies
MODS: Multiple Organs Dysfunction Syndrome
OR: Odds Ratio
PHIS: Pediatric Health Information System
PRISMA: Preferred Reporting Items for Systematic Reviews and Meta-Analyses
RCT: Randomized Controlled Trials
SVP: Single Ventricular Physiology
VAD: Ventricular Assist Devices.
