# Supplementary figures and images for: Use of Extracorporeal Membrane Oxygenation After Congenital Heart Disease Repair: A Systematic Review and Meta-Analysis
Source: Front Cardiovasc Med. 2020 Nov 11;7:583289. doi: 10.3389/fcvm.2020.583289 (PMC7686034; doi:10.3389/fcvm.2020.583289)

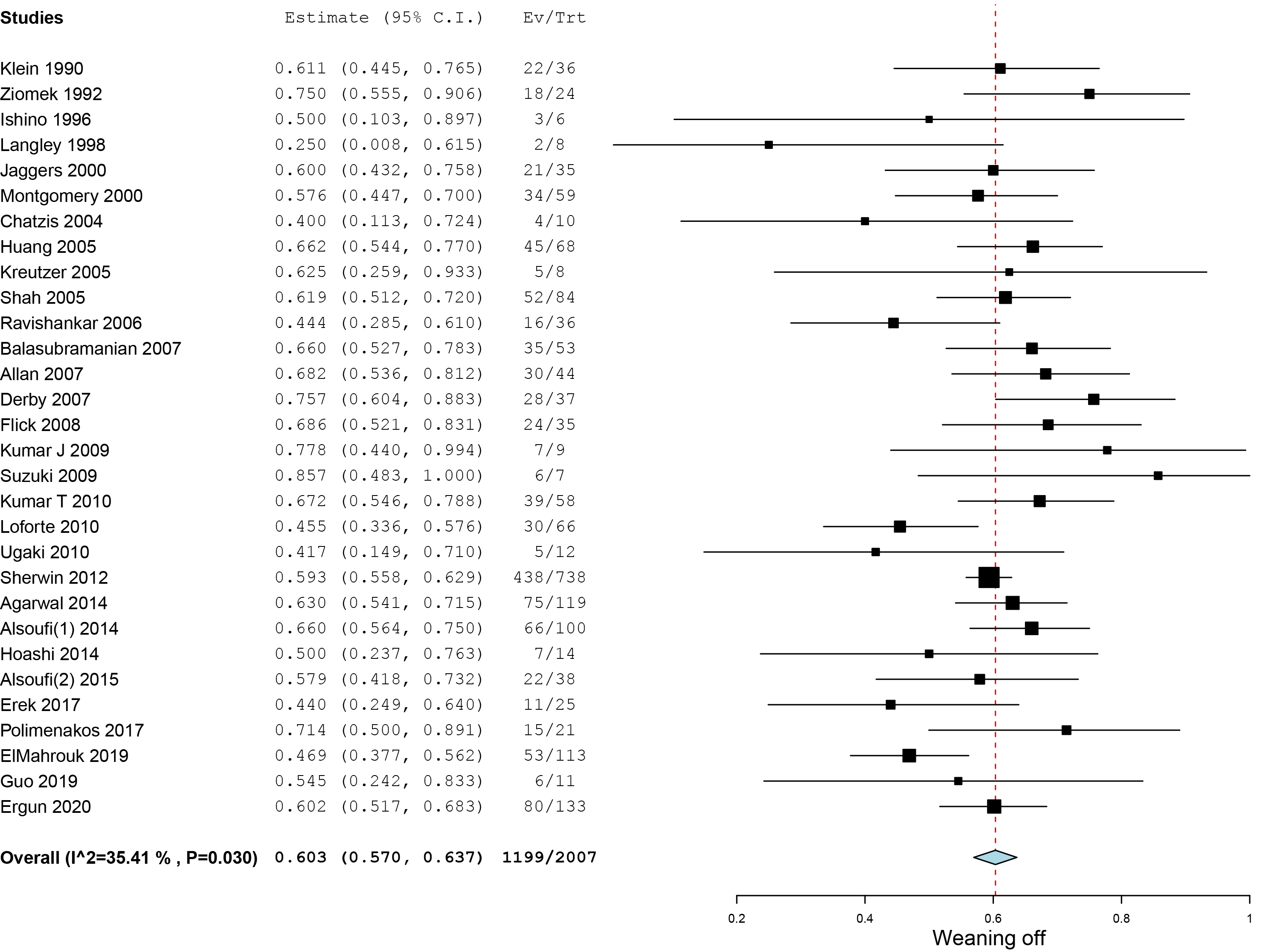

Supplement: Supplementary Figure 1 — Forest plot of incidence of successful weaning off in patients on extracorporeal membrane oxygenation. [file Image_1.TIF]

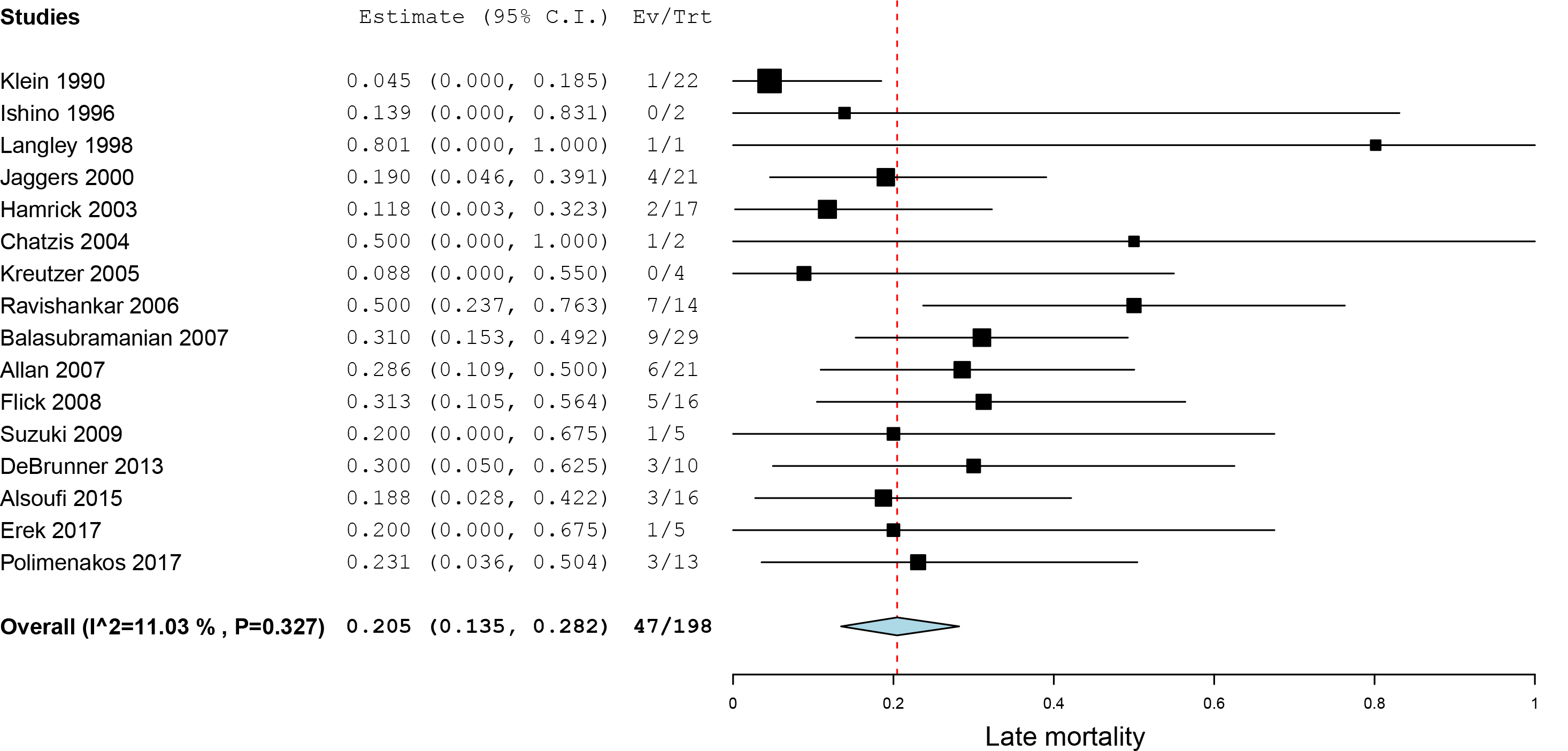

Supplement: Supplementary Figure 2 — Forest plot of incidence of late mortality in hospital survivors. [file Image_2.TIF]

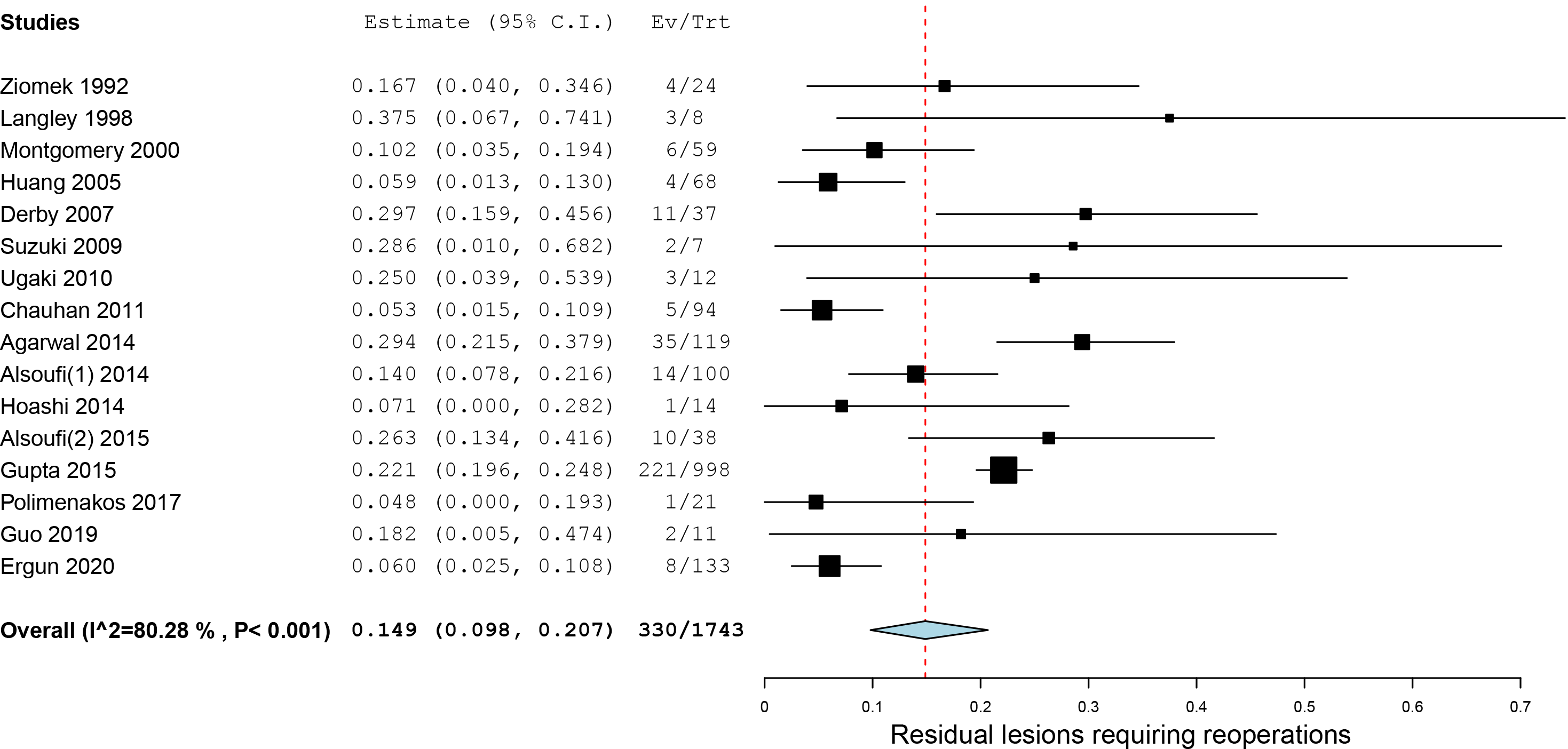

Supplement: Supplementary Figure 3 — Forest plot of incidence of residual lesions requiring reoperations in patients on extracorporeal membrane oxygenation. [file Image_3.TIF]

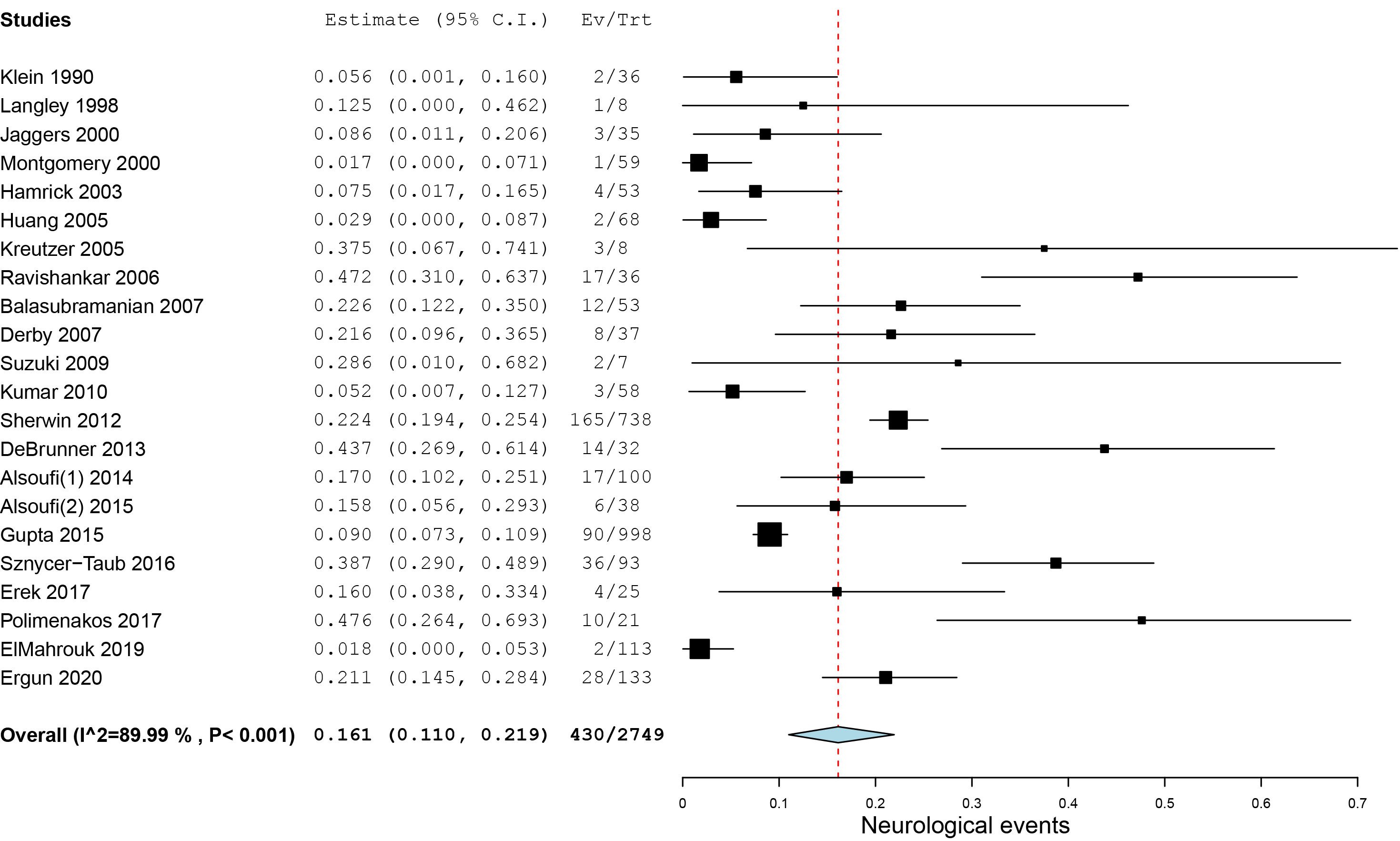

Supplement: Supplementary Figure 4 — Forest plot of incidence of neurological events in patients on extracorporeal membrane oxygenation. [file Image_4.TIF]

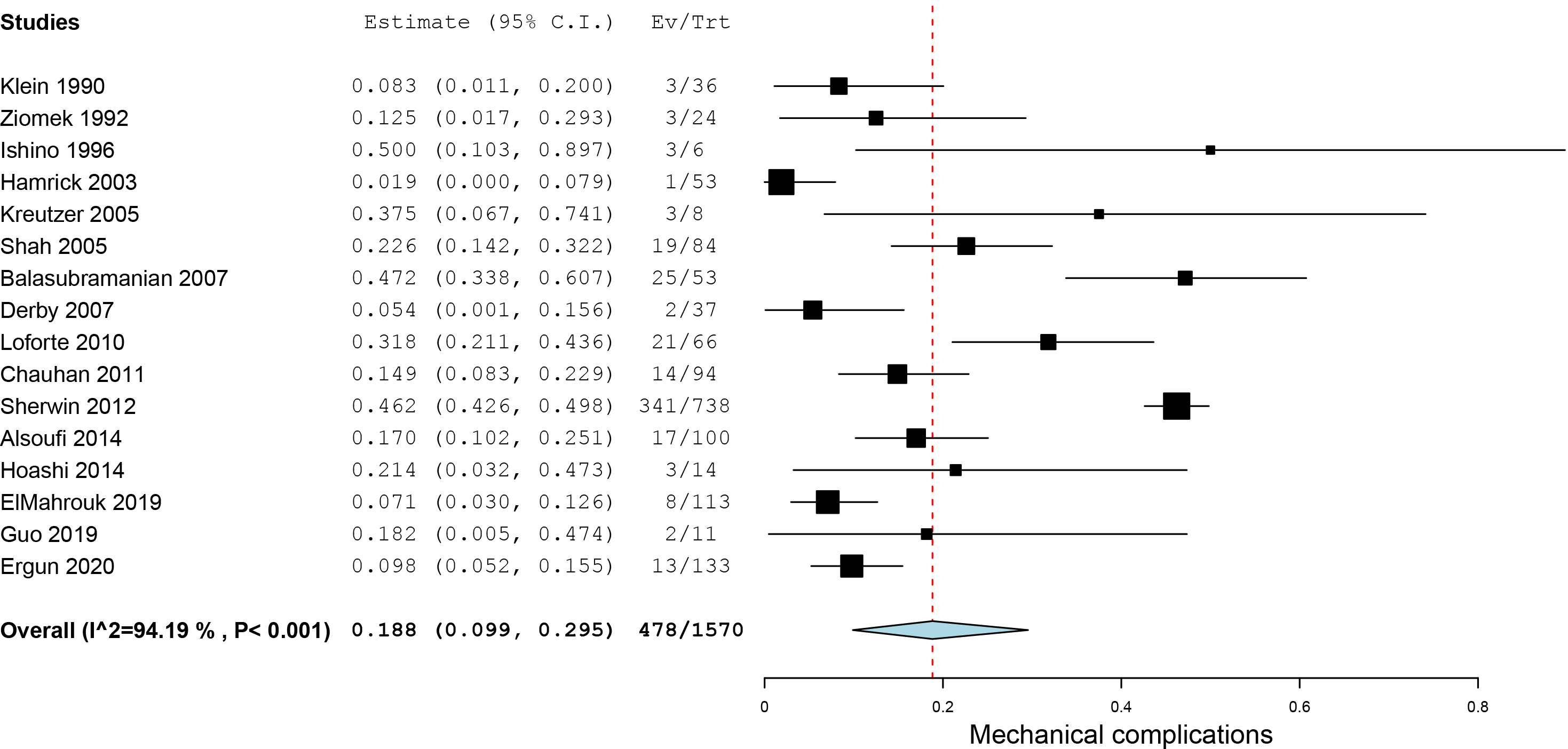

Supplement: Supplementary Figure 5 — Forest plot of incidence of ECMO mechanical complications in patients on extracorporeal membrane oxygenation. [file Image_5.TIF]

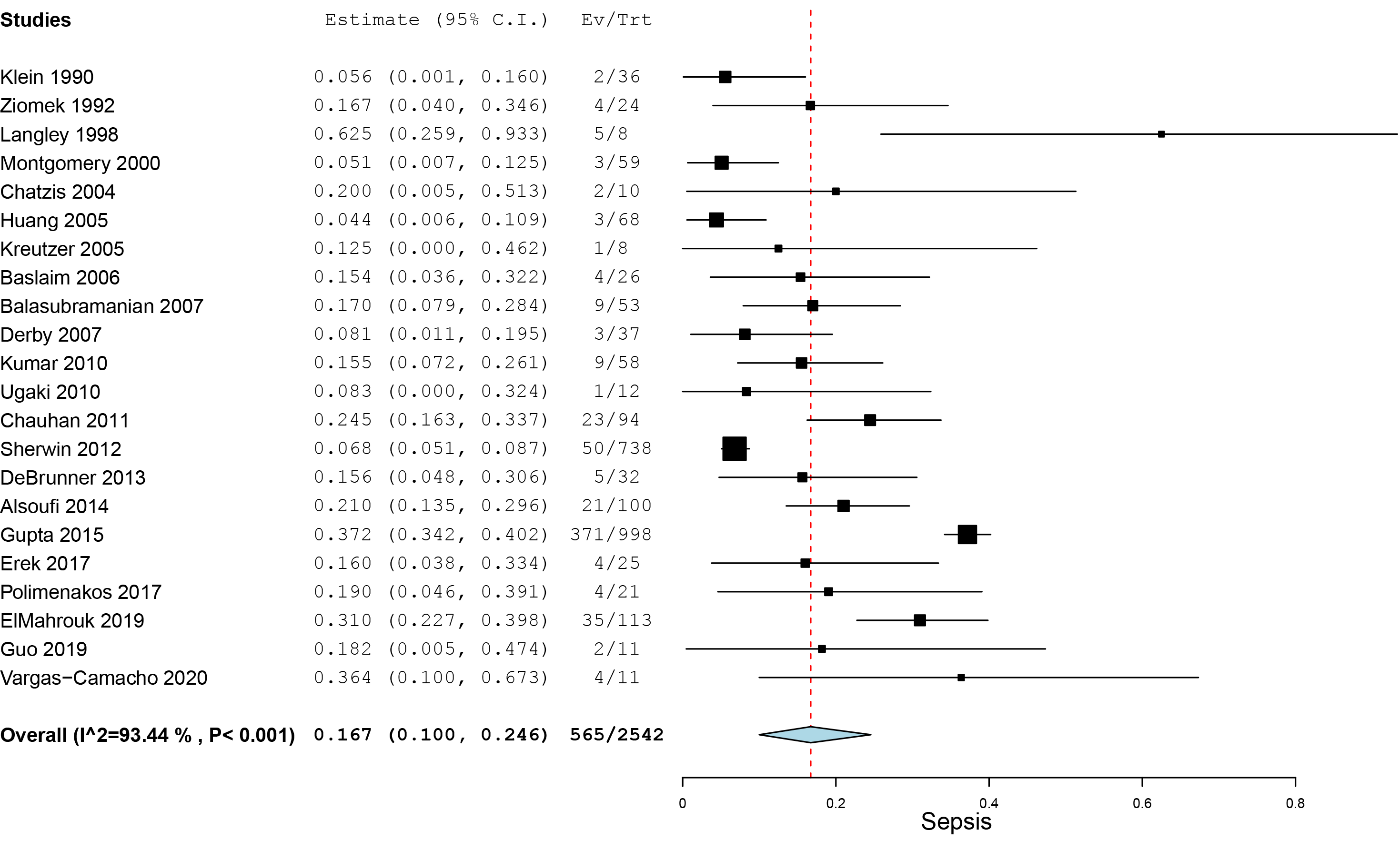

Supplement: Supplementary Figure 6 — Forest plot of incidence of sepsis in patients on extracorporeal membrane oxygenation. [file Image_6.TIF]

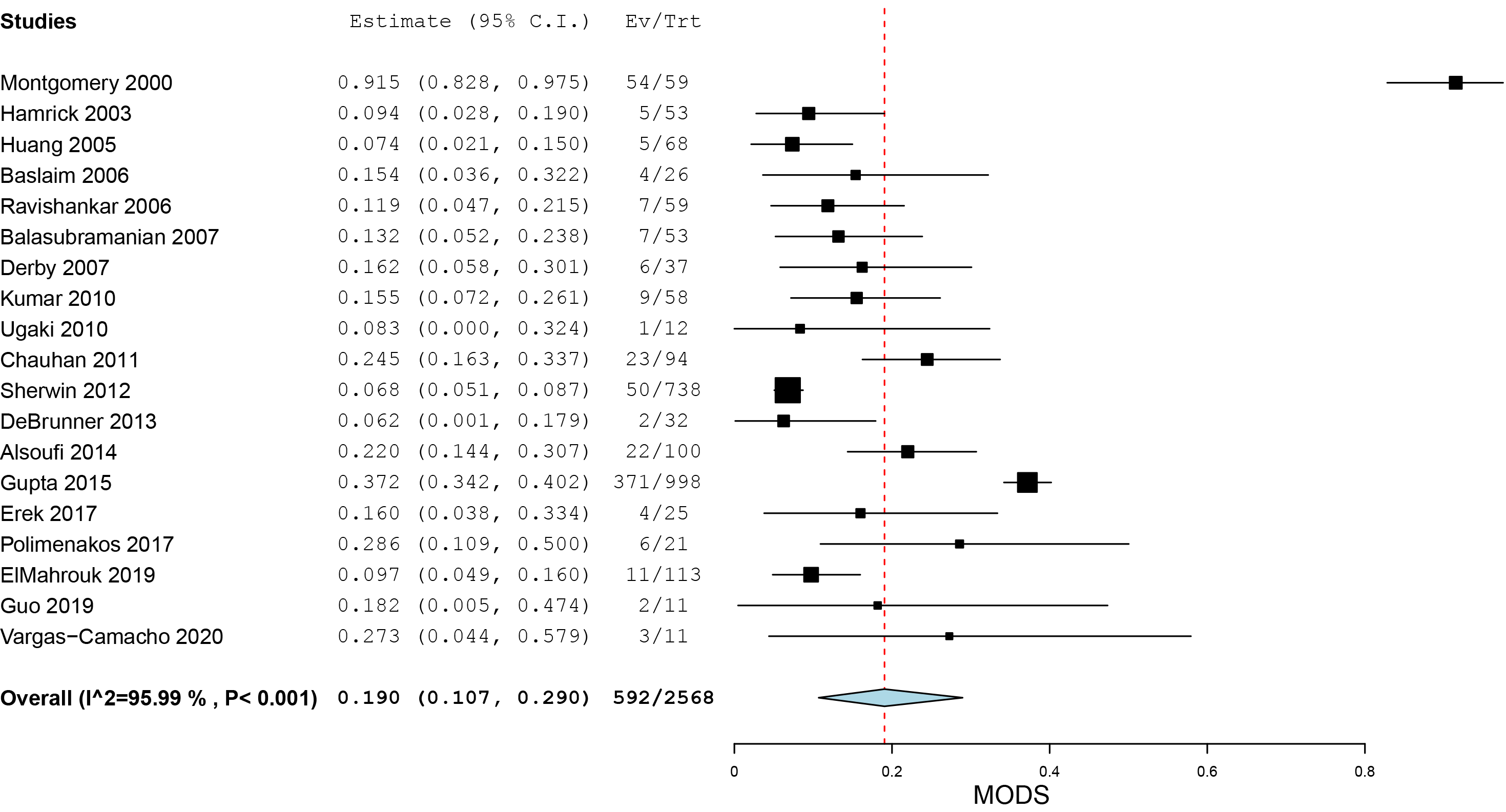

Supplement: Supplementary Figure 7 — Forest plot of incidence of MODS in patients on extracorporeal membrane oxygenation. [file Image_7.TIF]

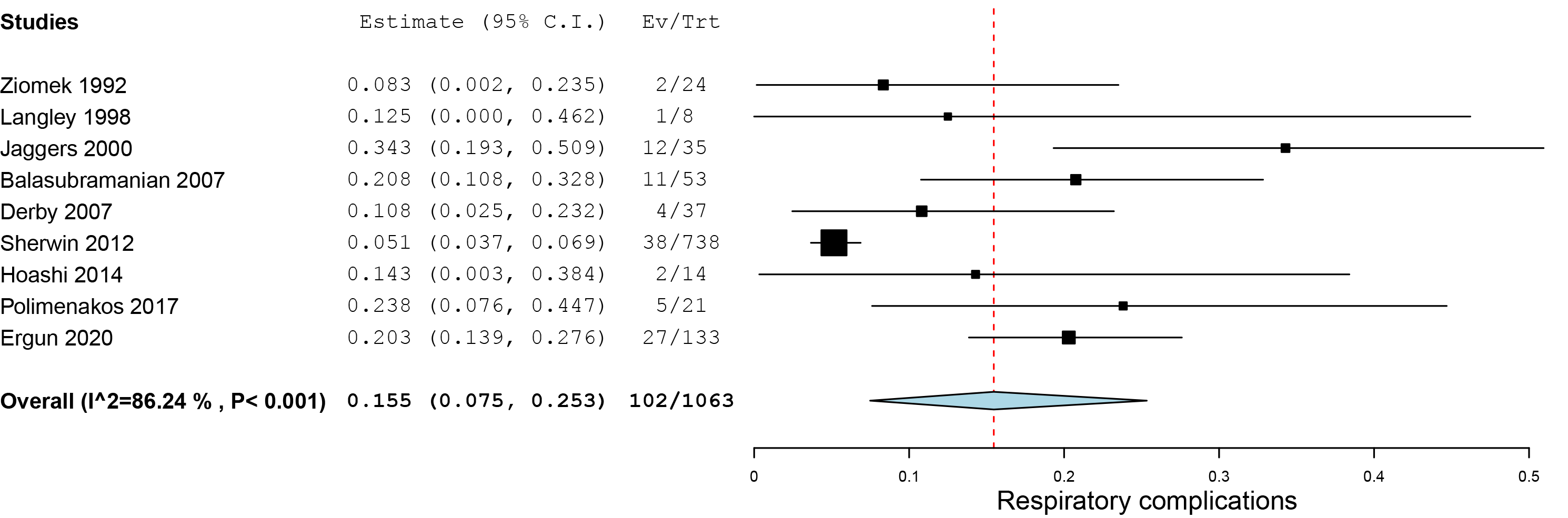

Supplement: Supplementary Figure 8 — Forest plot of incidence of respiratory complications in patients on extracorporeal membrane oxygenation. [file Image_8.TIF]
