# Supplementary material for: Use of Extracorporeal Membrane Oxygenation After Congenital Heart Disease Repair: A Systematic Review and Meta-Analysis
Source: Front Cardiovasc Med. 2020 Nov 11;7:583289. doi: 10.3389/fcvm.2020.583289 (PMC7686034; doi:10.3389/fcvm.2020.583289)
Supplement: Supplementary Table 1 — Baseline characteristics of included studies. [file Table_4.DOC]

**Supplemental Table 1** Baseline characteristics of included studies

| Author, year of publication, and area | Sample size  (Male/Female) | Age | Palliative surgery(%) | Cannulation at ICU/OR | ECMO indications* | ECPR  (%) | Cannulation site |
| --- | --- | --- | --- | --- | --- | --- | --- |
| Klein^[11]^, 1990  Detroit, USA | 36 | 13.6m(1d-7 y) | 11.1% | 29/7 | VF 30, PAH 6 | NA | Neck 30  Chest 6 |
| Ziomek^[12]^, 1992  Little Rock, USA | 12/12 | 12.5m(0.6d-6y) | 37.5% | 7/17 | VF 18, PAH 5,PF 1 | 4.2% | Neck 9  Chest 15 |
| Ishino ^[13]^, 1996  Boston, USA | 4/2 | 4y | 33.3% | NA | FW 6 | NA | Chest 6 |
| Langley^[14]^,1998  Southampton, UK | 6/2 | 8.6m | NA | 2/6 | VF 7, CA 1 | 12.5% | Chest 8 |
| Jaggers^[15]^, 2000  Durham, USA | 35 | 3m | 28.6% | 23/12 | VF 15, FW 10, CA 6 PAH 2,PF 2 | 17.1% | NA |
| Montgomery^[16]^, 2000  Louisville, USA | 59 | 6.4±10.2m | NA | 31/28 | VF 13，FW 21, CA 22,PF 3 | 42.3% | NA |
| Hamrick^[17]^, 2003  San Francisco, USA | 28/25 | 1-362d | NA | NA | VF 19, FW 21, CA 12, Arrhythmia 2 | 22.6% | NA |
| Chatzis^[18]^,2004  Athens, Greece | 6/4 | 3m(5d-28.5m) | 0 | NA | VF 7, FW 3 | NA | Chest |
| Huang^[19]^,2005  Taiwan, China | 41/27 | 1m(1d-14.7y) | 32.4% | 22/46 | VF 11, FW 46, CA 11 | 16.2% | Chest |
| Kreutzer^[20]^,2005  Buenos Aires, Argentina | 8 | 1m | 37.5% | NA | NA | NA | Chest |
| Shah^[21]^,2005  Nashville,USA | 84 | 4.3m | NA | 45\39 | FW 39, CA 28, VF 12, PAH 5 | 32.1% | Chest 79  Neck 5 |
| Baslaim^[22]^, 2006  Riyadh, Saudi Arabia | 26 | 16.4m(0.5-144m) | 23.1% | NA | VF 17,PF 6, Other 3 | 15.4% | Chest 26 |
| Ravishankar^[23]^, 2006  Philadelphia, USA | 26/10 | 1m | 100% | 20/16 | FW 14, CA 22 | 61.1% | Neck 20  Chest 16 |
| Balasubramanian^[24]^, 2007  Leicester, UK | 29/24 | 5m(1d-11y) | 7.5% | NA | VF 30, FW 13, CA 10 | 18.9% | Neck 42  Chest 11 |
| Allan^[25]^, 2007  Boston, USA | 44 | 8.0±2.3d | 100% | 37/7 | VF 22, CA 4, PF 15, PAH 1, Other 2 | 70.5% | NA |
| Derby^[26]^, 2007  Wilmington, USA | 37 | 9d(1d-1.3y) | 27% | 19/18 | VF 26, CA 6,PF 5 | 16.2% | Chest 37 |
| Flick^[27]^, 2008  Rochester, USA | 35 | 0-17y | NA | NA | NA | NA | NA |
| Kumar J^[28]^, 2009  Bangalore, India | 9 | 11±10m | 0 | NA | VF 5, CA 2, FW 2 | 22.2% | NA |
| Suzuki^[29]^, 2009  Aomori, Japan | 2/5 | 30m(2m-9y) | 57.1% | 3/4 | VF 3, PF 4 | NA | Neck/Femoral 2  Chest 5 |
| Kumar T^[30]^, 2010  Washington DC. USA | 31/27 | 0.4(0.1-7m) | 53.4% | NA | VF 8, CA 29, FW 19, PF 2 | 50% | Neck 10  Chest 48 |
| Loforte^[31]^, 2010  Berlin, Germany | 38/28 | 5.2±4y | 12.1% | NA | VF 17, FW 46, PAH 2, Arrhythmia 1 | 71.2% | NA |
| Ugaki^[32]^, 2010  Okayama, Japan | 8/4 | 1±1.1m | 100% | 6/6 | VF 6, CA 6 | 50% | Neck 1  Chest 11 |
| Chauhan^[33]^, 2011  New Delhi, India | 94 | 1.8m(1m-4y) | NA | 9/85 | VF 53, FW 22, PAH 10, Arrhythmia 9 | 1% | Chest 94 |
| McMullan^[34]^, 2011  Seattle, USA | 25 | 4.6m | 20% | 9/16 | VF 10, FW 8,PF 7 | NA | Neck 5  Chest 19 |
| Sherwin^[35]^, 2012  ELSO registry | 444/294 | 7(4–11)d | 100% | NA | VF 348, FW 209,PF 115 | 14.2% | Neck 172  Chest 566 |
| DeBrunner^[36]^, 2013  Pittsburgh, USA | 17/15 | <1m | 100% | NA | VF 9, FW 12, CA 11 | 34.4% | NA |
| Agarwal^[37]^, 2014  Nashville, USA | 67/52 | 0.5(0.3-2.7)m | 48.7% | 61/58 | VF 25, CA 34, FW 50, PAH 3,PF 5, Arrhythmia 2 | 28.6% | NA |
| Alsoufi^[38]^, 2014  Riyadh, Saudi Arabia | 63/37 | 2.5(0.2-194)m | 31% | 66/34 | NA | 37% | NA |
| Hoashi^[39]^, 2014  Osaka, Japan | 8/6 | 0.2-3.6m | 100% | 4/10 | VF 5, FW 3,PF 6 | NA | NA |
| Alsoufi^[40]^, 2015  Atlanta, USA | 21/17 | 0.2(0.1-0.3)m | 100% | NA | NA | 57.9% | NA |
| Gupta^[41]^, 2015  PHIS database | 562/436 | 0.5(0.1-218)m | NA | NA | NA | NA | NA |
| Sznycer-Taub^[42]^, 2016  Ann Arbor, USA | 54/39 | 7 (5-20)d | 37.6% | 51/42 | VF 42, FW 44, PAH 2, PF 2, SO 2 | 30.1% | Neck 13  Chest 80 |
| Erek^[43]^, 2017  İstanbul,Turkey | 18/7 | 3m(2d-4.5y) | 64% | NA | NA | 100% | Chest 25 |
| Polimenakos^[44]^, 2017  Oak Lawn, USA | 21 | 7.5±2.7d | 100% | NA | CA 17, PF 4 | 100% | NA |
| ElMahrouk^[45]^, 2019  Tanta, Egypt | 67/46 | 3m(4d-15 y) | 37.2% | 25/88 | VF 59, PF 34, CA 10 PAH 7, Arrhythmia 1, SO 1, Other 1 | 22.1% | Chest 113 |
| Guo^[46]^, 2019  Shanghai, China | 8/3 | 1.5(0.1-19)m | 9.1% | 9/2 | VF 5, PF 2, Residual lesions 4 | 100% | Chest 11 |
| Ergun^[47]^, 2020  Istanbul,Turkey | 66/67 | 4m (0.1m-13y) | 24.8% | NA | FW 44, PF 19, VF 41, CA 29 | 21.8% | Neck 20  Chest 113 |
| Vargas-Camacho^[48]^, 2020  Monterrey, Mexico9 | 7/4 | 5m | 18.2% | 5/6 | NA | NA | NA |
| Alsoufi^[49]^, 2005  Portland, USA | ECMO 16  VAD 13 | <1m | NA | NA | NA | NA | Chest 16 |
| Hoskote^[50]^, 2006  Toronto, Canada | ECMO 12  VAD 5 | 1.3±1.6m | 100% | 11/9 | VF 8, CA 8, SO 4 | 40% | Chest 20 |
| Kim^[51]^, 2015  Seoul, Korea | Conventional prime 25  Mini prime 39 | 14.3 ± 7.1d  13.5 ± 8.1 d | 16%  5.1% | NA | NA | NA | NA |
| Sasaki^[52]^,2014  Yokohama, Japan. | ECMO 24  VAD 12 | 64d ( 0–4.1 y) | 52.8% | NA | FW 14, VF 15, CA 7 | 19.4% | Chest |
| De Jesus-Brugman^[53]^, 2020  Indianapolis, USA | ECMO 25  No ECMO 60 | 9 (8-12)d | 100% | 5/20 | VF 4, FW 13, CA 5, PF 2, Arrhythmia 1 | 44% | NA |

*ECMO* Extracorporeal membrane oxygenation, *ECPR* Extracorporeal cardiopulmonary resuscitation, *ICU* Intensive care unit, *OR* Operating room, *VF* Ventricular failure, *PAH* Pulmonary artery hypertension, *FW* Failure to wean cardiopulmonary bypass, *SO* Shunt obstruction, *CA* Cardiac arrest, *PF* Pulmonary failure, *NA* Not available

*y* year, *m* month, *d* day

Data was expressed as mean value, or mean value±SD, or median value with range

*Ventricular failure involved left, right or bi-ventricular failure. Patients who received ECMO initiation due to hypoxaemia were categorized into pulmonary failure.
