# Supplementary material for: Use of Extracorporeal Membrane Oxygenation After Congenital Heart Disease Repair: A Systematic Review and Meta-Analysis
Source: Front Cardiovasc Med. 2020 Nov 11;7:583289. doi: 10.3389/fcvm.2020.583289 (PMC7686034; doi:10.3389/fcvm.2020.583289)
Supplement: Supplementary Table 2 — Types of involved congenital heart diseases and single ventricular physiology. [file Table_5.DOC]

**Supplemental Table 2** Types of involved congenital heart diseases and single ventricular physiology

| Author | Diagnosis | SVP (n) |
| --- | --- | --- |
| Klein^[11]^ | AVSD 10 VSD 8 TOF 4 DORV 4 TGA/ccTGA 4 ALCAPA 2 TAPVC 2 Other 2 | 4 |
| Ziomek^[12]^ | HLHS 4 TAPVC 3 TGA/ccTGA 3 AVSD PA 2 VSD 2 TOF 1 Other 6 | 9 |
| Ishino^[13]^ | AVSD 1 DORV 2 IAA 1 Other 1 | 1 |
| Langley^[14]^ | AVSD 3 TGA 2 TA 2 VSD 1 | NA |
| Jaggers^[15]^ | TOF 7 HLHS 5 DORV4 AVSD 3 IAA/COA 3 TAPVC 3 TGA/ccTGA 2 SVP 2 Other 6 | 8 |
| Montgomery^[16]^ | TOF 10 TGA 10 HLHS 8 TAPVC 7 AVSD 6 AS 3 ALCAPA 2 DORV 2 VSD 1 TA 1 Other 9 | NA |
| Hamrick^[17]^ | TOF 16 TAPVC 10 TGA/ccTGA 5 TA 5 HLHS 4 DORV 3 AVSD 2 IAA/COA 1 Other 7 | 8 |
| Chazis^[18]^ | TGA 6, DORV 1, ALCAPA 1, Other 2 | NA |
| Huang^[19]^ | NA | 22 |
| Kreutzer^[20]^ | HLHS 3, TGA 3, Other 2 | 3 |
| Shah^[21]^ | NA | 47 |
| Baslaim^[22]^ | TGA 18 AVSD 5 TAPVC 4 TA 3 PA 3 HLHS 2 DORV 2 Other9 | 6 |
| Ravishankar^[23]^ | HLHS 22 AVSD 9 TGA 1 Other 4 | 36 |
| Balasubramanian^[24]^ | TOF 16 TGA/ccTGA 10 VSD 6 TA 4 TAPVC 4 AVSD 4 SV 3 Other 6 | 3 |
| Allan^[25]^ | HLHS 24 PA 8 DORV 3 TGA1 TA1 Other 7 | 44 |
| Derby^[26]^ | HLHS 8 TGA 7 AVSD 5 AS 4 TOF 2 TA 2 TAPVC 2 PA 2 Other 5 | 10 |
| Flick^[27]^ * | RACHS-1 | NA |
| Kumar^[28]^ | VSD 5 TGA 2 TOF 2 | None |
| Suzuki^[29]^ | PA 3 TGA 1 AVSD 1 TOF 1 DORV 1 | 4 |
| Kumar^[30]^ | NA | 31 |
| Loforte^[31]^ | TGA 16 AS 6 DORV 6 AVSD 4 PA3 TOF 3 HLHS 2 TAPVC 2 Other 24 | *8* |
| Ugaki^[32]^ | HLHS 10 AS 2 | 12 |
| Chauhan^[33]^ | TGA 72 TOF 7 VSD 6 ALCAPA 4 AVSD 3 TA 2 | None |
| McMullan^[34]^* | RACHS-1 | NA |
| Sherwin^[35]^ | HLHS 738 | 738 |
| DeBrunner^[36]^ | HLHS 25 AVSD 5 Other2 | 32 |
| Agarwal^[37]^* | RACHS-1 | 58 |
| Alsoufi^[38]^* | RACHS-1 | 31 |
| Hoashi^[39]^ | HLHS\HLHS variants 14 | 14 |
| Alsoufi^[40]^ | HLHS 29 None-HLHS SVP 9 | 38 |
| Gupta^[41]^* | RACHS-1 | NA |
| Sznycer-Taub^[42]^# | STS-EACTS | 35 |
| Erek^[43]^ | TOF 5 HLHS 2 AS 1 TA 1 AVSD 1 TGA 1 Other 14 | NA |
| Polimenakos^[44]^ | HLHS14 None-HLHS SVP 7 | 21 |
| ElMahrouk^[45]^ | NA | 51 |
| Guo^[46]^ | COA 3 IAA 2 TAPVC 1 PA 1 ALCAPA 1 VSD 1 Other 2 | 1 |
| Ergün^[47]^ | HLHS 10 DILV 6 TA 6 DOLV 6 AVSD 6 PA 1, Other 98 | 33 |
| Vargas-Camacho^[48]^ | TGA 4 TOF 2 TAPVC 1 PS 1 VSD 1 PA 1 HLHS 1 | 2 |
| Alsoufi^[49]^ | NA | NA |
| Hoskote^[50]^ | HLHS 13 TGA 3 DORV 2 PA 1 Other 1 | 20 |
| Kim^[51]^ | RACHS-1 | NA |
| Sasaki^[52]^ | DORV 8 SV 7 HLHS 6 TGA 3 PA 3 TOF 1 AVSD 2 ALCAPA 1, Other 5 | 19 |
| De Jesus-Brugman^[53]^ | HLHS 20 AVSD 5 | 25 |

*HLHS* Hypoplastic left heart syndrome, *AVSD* Atrioventricular septal defect, *VSD* Ventricular septal defect, *TOF* Tetralogy of Fallot, *DORV* Double outlet of right ventricle, *DILV* Double inlet of left ventricle, *DOLV* Double outlet of left ventricle, *TGA/ccTGA* Transposition of the great arteries, *ALCAPA* Anomalous left coronary artery originating from the pulmonary artery, *TAPVC* Total anomalous pulmonary venous connection, *PA* Pulmonary artery atresia, *TA* Truncs arteriosus, *CoA* Coarctation of the aorta, *IAA* Interrupted aortic arch, *RACHS* Risk Adjustment for Congenital Heart Surgery classification, *STS-EACTS* Society of thoracic surgeons–European association for cardiothoracic surgery congenital heart surgery database category

* Congenital heart diseases were categorized in *RACHS* classification

# Congenital heart diseases were categorized in STS-EACTS classification
