# Supplementary material for: Use of Extracorporeal Membrane Oxygenation After Congenital Heart Disease Repair: A Systematic Review and Meta-Analysis
Source: Front Cardiovasc Med. 2020 Nov 11;7:583289. doi: 10.3389/fcvm.2020.583289 (PMC7686034; doi:10.3389/fcvm.2020.583289)
Supplement: Supplementary Table 3 — Meta-regression analysis for in-hospital mortality based on pre-specified patients' variables. [file Table_6.DOC]

**Supplemental Table 3** Meta-regression analysis for in-hospital mortality based on pre-specified patients’ variables

| **Variables** | **Univariate meta-regression** | | | **Multivariate meta-regression** | | |
| --- | --- | --- | --- | --- | --- | --- |
|  | **Coefficient** | **95% Confidence interval** | **p value** | **Coefficient** | **95% Confidence interval** | **p value** |
| CPB (min) | 0.001 | 0.000 to 0.0028 | 0.179 | - | - | - |
| ACC (min) | -0.001 | -0.003 to 0.002 | 0.455 | - | - | - |
| Age (months) | -0.002 | -0.006 to 0.002 | 0.225 | - | - | - |
| Weight (Kg) | -0.011 | -0.032 to 0.008 | 0.252 | - | - | - |
| Peak lactate (mmol/L) | -0.008 | -0.026 to 0.009 | 0.316 | - | - | - |
| Lowest arterial PH | 0.338 | -1.166 to 1.842 | 0.612 | - | - | - |
| **SVP (%)** | **0.169** | **0.053 to 0.286** | **0.006^*^** | **0.213** | **0.099 to 0.327** | **0.001^*^** |
| Cannulation at OR(%) | -0.014 | -0.361 to 0.333 | 0.933 | - | - | - |
| ECPR (%) | 0.056 | -0.145 to 0.258 | 0.572 | - | - | - |
| Bleeding (%) | 0.003 | -0.246 to 0.253 | 0.979 | - | - | - |
| **Renal failure(%)** | **0.343** | **0.117 to 0.568** | **0.004^*^** | **0.315** | **0.091 to 0.540** | **0.008^*^** |
| Sepsis(%) | -0.018 | -0.531 to 0.495 | 0.943 | - | - | - |
| Stroke(%) | -0.625 | -0.527 to 0.402 | 0.780 | - | - | - |
| MODS(%) | -0.076 | -0.392 to 0.241 | 0.620 | - | - | - |
| HLHS(%) | 0.127 | -0.041 to 0.295 | 0.133 |  |  |  |
| **ECMO duration(hours)** | **0.001** | **0.000 to 0.001** | **0.031^*^** | **0.000** | **-0.000 to 0.001** | **0.593** |

*SVP* Single ventricular physiology, *OR* Operating room, *ECPR* Extracorporeal cardiopulmonary resuscitation, *MODS* Multiple organs dysfunction syndrome, *ECMO* Extracorporeal membrane oxygenation

* P<0.05
