# Supplementary material for: Use of Extracorporeal Membrane Oxygenation After Congenital Heart Disease Repair: A Systematic Review and Meta-Analysis
Source: Front Cardiovasc Med. 2020 Nov 11;7:583289. doi: 10.3389/fcvm.2020.583289 (PMC7686034; doi:10.3389/fcvm.2020.583289)
Supplement: Supplementary file 13 [file Table_2.DOC]

**Excluded studies in the full-text evaluation process**

| **Review, case report,case series<5 cases** | **Year of publication** |
| --- | --- |
| Ferrazzi | 1991 |
| Dudell | 1993 |
| Plowden | 1991 |
| O'Connor | 1993 |
| Alexi-Meskishvili | 1994 |
| Collison | 2006 |
| Salvin | 2008 |
| Ye | 2010 |
| Brown | 2012 |
| Hraska | 2014 |
| Thiagarajan | 2016 |
| Alexander | 2017 |
| **Bridge to transplant** | |
| Meliones | 1991 |
| Mitchell | 2000 |
| **Non-CHD conditions** | |
| Butt | 1992 |
| del Nido | 1992 |
| Palmisano | 1992 |
| Raithel | 1992 |
| del Nido | 1994 |
| Seib | 1999 |
| Tanke | 2002 |
| Kolovos | 2003 |
| Chaturvedi | 2004 |
| Chow | 2004 |
| Morris | 2004 |
| Undar | 2004 |
| Hintz | 2005 |
| Hannan | 2006 |
| Alsoufi | 2007 |
| Chan, | 2008 |
| Lequier | 2008 |
| Zhao | 2008 |
| Kane | 2010 |
| Itoh | 2011 |
| Wolf | 2012 |
| Chrysostomou | 2013 |
| De Rita | 2014 |
| Garcia Guerra | 2014 |
| Mascio | 2014 |
| Merrill | 2014 |
| Turek | 2013 |
| Philip | 2014 |
| Flórez | 2015 |
| Polito | 2015 |
| Botha | 2016 |
| Ford | 2016 |
| Shin | 2016 |
| Elias, | 2017 |
| Brunetti | 2018 |
| Chen | 2018 |
| Mistry | 2018 |
| Shah | 2018 |
| Achuff | 2019 |
| Sadhwani | 2019 |
| Black | 1995 |
| Aharon | 2001 |
| Miana | 2015 |
| **ECMO prior to CHD repair** | |
| Faulkner | 1994 |
| Dhillon | 1995 |
| Khan | 1996 |
| Stewart | 1996 |
| Pizarro | 2001 |
| Bae | 2005 |
| McKenzie | 2017 |
| Delius | 1992 |
| Walters | 1995 |
| Duncan | 1998 |
| Jacobs | 2000 |
| Misfeldt | 2015 |
| **Duplicates** | |
| Kulik | 1996 |
| McKay | 1997 |
| Ibrahim | 2000 |
| Mahle | 2005 |
| Pizarro | 2006 |
| Thourani | 2006 |
| Alsoufi | 2009 |
| Nardell | 2009 |
| Imamura | 2011 |
| Shuhaiber | 2011 |
| Alsoufi | 2014 |
| Friedland-Little | 2014 |
| Gupta | 2014 |
| Jolley | 2014 |
| Peer | 2014 |
| Peer | 2014 |
| Gupta | 2015 |
| Aydin, | 2016 |
| Friedland-Little | 2017 |
| Furlong-Dillard | 2017 |
| Gomez | 2017 |
| Eghbalzadeh | 2018 |
| Polimenakos | 2011 |
| **Patients over 18 years of age were involved** | |
| Trittenwein | 1999 |
| Fiser | 2003 |
| Ghez | 2005 |
| Erek | 2013 |
| Pilan | 2019 |
| **ECMO without the oxygenator** | |
| Darling | 2001 |
| **Outcomes of not interest** | |
| Agati | 2006 |
| Dipchand | 2015 |
| Burke | 2017 |
| Jegatheeswaran | 2020 |
| Coskun | 2010 |
