# Supplementary material for: Use of Extracorporeal Membrane Oxygenation After Congenital Heart Disease Repair: A Systematic Review and Meta-Analysis
Source: Front Cardiovasc Med. 2020 Nov 11;7:583289. doi: 10.3389/fcvm.2020.583289 (PMC7686034; doi:10.3389/fcvm.2020.583289)
Supplement: Supplementary file 14 [file Table_3.DOC]

**Pubmed search strategy**

**Congenital heart diseases**

#1((("congenital"[Subheading] OR "congenital"[All Fields]) AND ("heart diseases"[MeSH Terms] OR ("heart"[All Fields] AND "diseases"[All Fields]) OR "heart diseases"[All Fields])) OR ("heart defects, congenital"[MeSH Terms] OR ("heart"[All Fields] AND "defects"[All Fields] AND "congenital"[All Fields]) OR "congenital heart defects"[All Fields] OR ("congenital"[All Fields] AND "heart"[All Fields] AND "defects"[All Fields])) OR (("congenital"[Subheading] OR "congenital"[All Fields]) AND ("heart"[MeSH Terms] OR "heart"[All Fields]) AND ("abnormalities"[Subheading] OR "abnormalities"[All Fields] OR "anomalies"[All Fields])) OR ("heart defects, congenital"[MeSH Terms] OR ("heart"[All Fields] AND "defects"[All Fields] AND "congenital"[All Fields]) OR "congenital heart defects"[All Fields] OR ("congenital"[All Fields] AND "cardiac"[All Fields] AND "defects"[All Fields]) OR "congenital cardiac defects"[All Fields]) OR (("congenital"[Subheading] OR "congenital"[All Fields]) AND ("heart diseases"[MeSH Terms] OR ("heart"[All Fields] AND "diseases"[All Fields]) OR "heart diseases"[All Fields] OR ("cardiac"[All Fields] AND "diseases"[All Fields]) OR "cardiac diseases"[All Fields])))

**Extracorporeal membrane oxygenation**

#2 (("extracorporeal membrane oxygenation"[MeSH Terms] OR ("extracorporeal"[All Fields] AND "membrane"[All Fields] AND "oxygenation"[All Fields]) OR "extracorporeal membrane oxygenation"[All Fields] OR "ecmo"[All Fields]) OR ECLS[All Fields] OR ("extracorporeal membrane oxygenation"[MeSH Terms] OR ("extracorporeal"[All Fields] AND "membrane"[All Fields] AND "oxygenation"[All Fields]) OR "extracorporeal membrane oxygenation"[All Fields]) OR (Extracorporeal[All Fields] AND ("lung"[MeSH Terms] OR "lung"[All Fields]) AND support[All Fields]) OR ("extracorporeal membrane oxygenation"[MeSH Terms] OR ("extracorporeal"[All Fields] AND "membrane"[All Fields] AND "oxygenation"[All Fields]) OR "extracorporeal membrane oxygenation"[All Fields] OR ("extracorporeal"[All Fields] AND "life"[All Fields] AND "support"[All Fields]) OR "extracorporeal life support"[All Fields]))

**Search #1 AND #2**

Filter: English,Year of publication 1990-2020
